# Successful nonoperative management of high output enterocutaneous fistulae in high surgical risk HIV‐positive patients: Two case reports and literature review

**DOI:** 10.1002/ccr3.1840

**Published:** 2018-10-30

**Authors:** David Muchuweti, Edwin Gamba Muguti, Simbarashe Gift Mungazi

**Affiliations:** ^1^ Department of Surgery, College of Health Sciences University of Zimbabwe Harare Zimbabwe; ^2^ College of Health Sciences University of Zimbabwe Harare Zimbabwe; ^3^ Department of Surgery and Anaesthetics, Faculty of Medicine National University of Science and Technology Bulawayo Zimbabwe

**Keywords:** enterecutaneous fistulae, high output, human immunodeficiency virus infection, hypoalbuminemia, malnutrition, sepsis

## Abstract

Management of enterocutaneous fistulae is challenging, often requiring a multidisciplinary approach. In high output fistulae, surgery is advocated after control of sepsis, adequate fluid and electrolyte repletion, and nutritional support. Surgery may, however, be contraindicated in the presence of sepsis and malnutrition. The presence of HIV infection brings extra challenges.

## 1 INTRODUCTION

Management of enterocutaneous fistulae is challenging, often requiring a multidisciplinary approach. In high output fistulae, surgery is advocated after control of sepsis, adequate fluid and electrolyte repletion, and nutritional support. We present two high surgical risk HIV‐positive patients with high output enterocutaneous fistulae, we successfully managed nonoperatively.

An enterocutaneous fistula (ECF) is an abnormal communication between the gastrointestinal tract and the skin. The majority 75%‐85% are as a result of iatrogenic trauma during bowel surgery. The ileum is the commonest site of the fistulae.^1^ The remainder of the fistulae occur spontaneous as a result of irradiation, malignancy, human immunodeficiency virus (HIV)^2^ infection among other causes. HIV infection is associated with chronic ileitis and cytomegalovirus infection of the bowel, both conditions which predisposes to bowel perforation.^3^ HIV infection is a nutritionally progressive disorder with major metabolic changes in nutrient utilization. Due to its chronicity, HIV infection is associated with malnutrition which typically presents with hypoalbuminemia.^4^, ^5^ In HIV‐positive patients with ECF, the malnutrition brought about by HIV infection imparts negatively on both nonoperative and operative management of patients with ECF resulting in high morbidity and mortality. The immunologic abnormalities associated with chronic HIV disease predispose patients to systemic bacterial infections.^6^, ^7^

Classification of ECF by anatomic, physiologic, and etiology is critical to both nonoperative and operative treatment planning.^8^ Anatomically, fistulae can be proximal or distal and may be classified based on the part of the gastrointestinal tract involved.^8^ Physiologic classification is based on fistula output. Fistulae with a daily output of <200 mL/d being classified as low output fistulae and those above 500 mL/d high output fistulae those between 200 and 500 mL/d as intermediate output. Fistula output is a significant single prognostic factor for determining the possibility of spontaneous closure and mortality.^9^, ^10^

Management involves fluid and electrolytes resuscitation, treatment of sepsis, nutritional support, wound care, and effluent control. Spontaneous closure of ECF with nonoperative management is well documented with 90% of spontaneous closure occurring in the first month after sepsis resolution and additional 10% closing in the second month.^11^, ^12^ Closure rates are higher with low output than high output fistulae. Fistulae not closed by 2 months are unlikely to close^12^ and will need surgery. The use of Somatostatin and its analogues reduces gastrointestinal secretions, and hence, fistula output and in some studies has been shown to help speed up fistula closure.^13^ Failed medical treatment, high output fistulae, and fistula associated with diseased bowel, distal obstruction, or eversion of mucosa are some of the indications for surgery. Surgery involves takedown of the fistulous tract and resection of the bowel involved followed by primary anastomosis. Surgical therapy should be undertaken in patients with no adverse factors such as sepsis, malnutrition, hypoalbuminemia.^14^ Surgical therapy will fail in the presence of adverse effects.^14^ Our patients had adverse effects which precluded surgical management. We present the two patients below.

## 2 CASE 1

A 35‐year‐old, Mrs MM, a widowed HIV‐positive patient on antiretroviral therapy (ART) for 6 years presented to a general surgical unit with abdominal pain and distension, vomiting, foul‐smelling vaginal discharge, and fever for a week. She has one child and lost the second child who was aged 8 months from HIV‐related pneumonia. On examination, she was ill‐looking, pale, and pyrexial. Her temperature and pulse rate were 38°C and 125 beats/min, respectively. Her blood pressure was 112/68. Her abdomen was distended but there was no guarding and rebound tenderness. She had deep sited tenderness in the left and right lower quadrants and in the suprapubic region. There was hyper‐resonant percussion note and with decreased bowel sounds. There was tenderness on digital rectal examination. On vaginal examination, there was cervical excitation tenderness with a thick white foul smelling discharge on glove. Other systems were normal. A clinical diagnosis of pelvic abscess was made, and a gynecological consultation was made. A joint laparotomy was planned.

Her preoperative investigations included a chest X‐ray and erect and supine abdominal X‐rays. The chest X‐ray showed air under the right hemidiaphragm and abdominal X‐rays showed distended loops of small bowel. Her full blood count (FBC) showed an elevated white cell count of 13.0 × 10^9^, a low hemoglobin of 9.6 g/dL and a platelet count of 606 × 10^9^ cells/L. Sodium and Potassium were normal and were 140 and 4.4 mmol/L, respectively. Urea was raised to 9.8 mmol/L. Creatinine was 46 mmol/L. She had a low albumen and total protein of 22 and 67 g/dL, respectively. Her CD4 cell count was 230 cells/L.

Fluid resuscitation was commenced. A transurethral catheter and nasogastric tube were inserted. The patient was commenced on Ceftriaxone 1 g daily and Metronidazole 500 mg 8 hourly intravenously. Following good resuscitation, she was taken to theater for a laparotomy. At laparotomy, loops of small bowel matted by adhesions were noted. Adhesionolysis was done to free loops of small bowel. Approximately 2500 mL of pus were drained from the pelvis. Peritoneal lavage was done using 10 L of warm normal saline. An inadvertent enterotomy was noted intraoperatively and repaired with 3/0 PDS. The abdominal incision was closed in two layers with 0/PDS for the sheath and 2/0 Nylon for the skin. Intraoperatively the patient went into septic shock and required inotropic support. She was put on Dopamine infusion 4 mL/h.

Postoperatively the patient was admitted in the intensive care unit (ICU) for high care. Antibiotics, analgesia, fluid management, and monitoring were continued in ICU. By Day 3, the patient had shown much improvement. She was extubated and discharged to high care unit (HCU). Whilst in HDU on day 4, the patient was noted to be leaking fluid from the suture line. Fecal matter was noted when two skin sutures were removed. A colostomy bag was applied and the initial drainage noted on day 5 was 900 mL. A diagnosis of a high output enterocutaneous (ECF) fistula was made. Her white cell count went up to 21 × 10^9^/fl. Fluid management, correction of electrolyte abnormalities, nutritional support, antibiotic management, skin protection, and monitoring and charting fistula daily fistula output were prioritized. Electrolytes were measured every other day. The following table (Table 1) shows summary of results and progress of the patient until discharge on day 31:

**Table 1:** Summary of results and progress of the patient until discharge on day 31

Result	Day 5 post op	Day 6	Day 7	Day 9	Day 11	Day 15		Day 20	Day 25	Day 30	Day 31
Na (mmol/L)	140	141	146	143	136	139		136	132	132	
K (mmol/L)	4.2	4.1	3.9	4.2	3.3	3.5		4.7	4	4	
Urea (mmol/L)	4.8	4.7	6.4	4.1	1.3	1.1		2.1	2.6	3.1	
Creatinine (mmol/L)	40	46	43	34	92	67		33	29	28	
WCC (µmol/L)	21	19	15	15.1	14.8	13.2		11.24	9.29	7	8
Hb (g/dL)	9.8	9.4	10.4	9.7	9.9	9.2		9.1	7.8		
Plt (ft)	322	296	951	690	650	389		380	390		
ph	7.42										
Po_2_	96										
Pco_2_	37.3										
Bicarbonate	25										
SPo_2_ (%)	96	95									
Alb (g/dL)	15		15	15	19	19		20	20	22	
Tp (g/dL)	63		49	47		56		66	65		
IV daily fluid intake (mL)	4070	4471	3200	3000	4140	3500		2800			
Urine output (mL)	2370	2070	1500	1700	4500	1500		2000			
Temperature (°C)	37.5	36	36.9	36	37	36		37			36
Fistula output (mL)							900				970	1880	1300	650	300	229	30	5	0
Diet‐high protein high calorie diet (HPHC)							HPHC				HPHC	HPHC	HPHC	HPHC	HPHC	HPHC	HPHC	HPHC	HPHC

## 3 CASE 2

A 28‐year‐old HIV‐positive Mr EM on ART for the past 2 years was admitted with a 2‐day history of abdominal distension, vomiting, and a fever. Three days prior to admission, he was being treated for diarrhea with oral rehydration fluids and Cotrimoxazole at a local clinic. He was referred to hospital because his condition was not getting better.

On examination, he was ill‐looking and in respiratory distress. He had tachycardia and tachypnea with a pulse rate and respiratory rate of 110 beats/min and 30 breaths/min, respectively. His arterial oxygen saturation on free air was 89%. He had a temperature of 38°C and a blood pressure of 100/70. The abdomen was distended and moving minimally with respiration. Guarding, tenderness, and rebound tenderness were elicited on palpation. Bowel sounds were diminished. Other systems were normal.

A diagnosis of secondary peritonitis secondary to a perforated duodenal ulcer was made. Fluid resuscitation was commenced. The patient was propped up in bed and put on oxygen per face mask. Saturation improved to 94%. Ceftriaxone 1 g/d and Metronidazole 500 mg intravenously three times a day were commenced. A nasogastric tube for free drainage was inserted and a transurethral catheter was inserted to monitor urine output. Pethidine 100 mg 6 hourly was given for analgesia. The following were his preoperative laboratory investigations: Full blood count: Hb = 10 g/dL, WCC = 25 000 µmol/L Platelets = 190 × 10^3^. Urea and electrolytes were normal. His preoperative albumin was low, 18 g/dL. His CD4 cell count was 350 cells/L.

The patient was taken to theater for laparotomy the same day in the evening because of worsening respiratory distress. At laparotomy, it was noted that the patient had multiple adhesions. Two small bowel perforation, each approximately 1 cm in diameter 2 cm apart and 25 cm from the ileocecal valve were noted. There was ascites and fecal contamination of the peritoneal cavity. There was no perforated duodenal ulcer. Ascitic fluid was suctioned out, and segment of small bowel with two perforations was resected followed by end‐to‐end small bowel anastomosis. Peritoneal lavage was done using 4 L of warm saline and the abdomen was closed in layers.

The patient was admitted in high dependence unit (HDU). Fluid management, oxygen per face mask and antibiotics were continued in HDU. Clexane 40 mg subcutaneously daily and chest physiotherapy were commenced. On day three postoperation, the patient was discharged to a general surgical ward. On the sixth day, the patient’s abdomen began to distend and he developed a fever of 37.9°C. A discharge was noted from the suture line and on removal of two skin sutures fecal material was noted coming out. A colostomy bag was applied and over 24 hours a fistula output of 1500 mL was recorded. A diagnosis of high output enterocutaneous fistula was made. Antibiotics, intravenous iv fluid were continued and a high calorie high protein diet was commenced. The following are the serial results and progress of the patient shown in Table 2 until discharge on day 46.

**Table 2:** Showing results and patient’s progress

Result	Day 5 post op	Day 6	Day 7	Day 9	Day 11	Day 15	Day 20	Day 25	Day 30	Day 31
Na (mmol/L)	140	141	146	143	136	139	136	132	132		
K (mmol/L)	4.2	4.1	3.9	4.2	3.3	3.5	4.7	4	4		
Urea (mmol/L)	4.8	4.7	6.4	4.1	1.3	1.1	2.1	2.6	3.1		
Creatinine (mmol/L)	40	46	43	34	92	67	33	29	28		
WCC (µmol/L)	21	19	15	15.1	14.8	13.2	11.24	9.29	7	8	
Hb (g/dL)	9.8	9.4	10.4	9.7	9.9	9.2	9.1	7.8			
Plt × 10^9^ (ft)	322	296	951	690	650	389	380	390			
PH	7.42										
P02	96										
Pco_2_	37.3										
Bicarbonate	25										
SPo_2_ (%)	96	95									
Alb (g/dL)	15		15	15	19	19	20	20	22		
Tp (g/dL)	63		49	47		56	66	65			
IV daily fluid intake (mL)	4070	4471	3200	3000	4140	3500	2800				
Urine output (mL)	2370	2070	1500	1700	4500	1500	2000				
Temperature (°C)	37.5	36	36.9	36	37	36	37			36	
Fistula output (mL)	900	970	1880	1300	650	300	229	30	5	0	
Diet‐high protein high calorie diet (HPHC)	HPHC	HPHC	HPHC	HPHC	HPHC	HPHC	HPHC	HPHC	HPHC	HPHC	

## 4 DISCUSSION

Enterocutaneous fistula (ECF) is the most feared complication of abdominal surgery because of the associated morbidity and mortality rates. High mortality rates of 25.4% have been reported,^15^ mainly from sepsis, malnutrition, and electrolyte imbalance. The etiology of ECF in HIV infected patients is similar to the noninfected patients. Iatrogenic trauma during abdominal surgery is by far the most common cause of ECF^16^, ^17^ though spontaneous fistula has been reported in HIV infected patients.^2^ The majority of fistula follow emergency abdominal surgery.^18^, ^19^ Both our patients had HIV infection with iatrogenic trauma during emergency abdominal surgeries as the cause of fistulae and the fistulae were high output fistulae.

The diagnosis of ECF is usually obvious with external drainage of enteric contents. Typically, ECF presents postoperatively on day 5 or 6 with abdominal pain, fever, and prolonged ileus followed by drainage of fluid from the suture line.^15^ A diagnosis of surgical site infection is initially made but removal of one or two sutures results in the drainage of enteric contents. Work up involves laboratory and imaging investigations. Laboratory investigations include full blood count, urea, and electrolytes, and blood gases. A CT scan and a fistulogram will help define the anatomy of the fistula and detect any other intra‐abdominal pathology. Once the diagnosis of ECF has been made, there is need for controlled drainage of the fistula. This helps to determine the output of the fistulae which has a bearing on management. Proximal fistulae are likely to be high output fistulae.^20^ High output fistulae have a daily output of 500 mL and above (7.8) and a significant number do not close with nonoperative management.^20^ Our patients had high output fistulae which potentially required early surgery.

Complications of enterocutaneous fistula include sepsis, fluid and electrolyte derangements, malnutrition, and skin excoriation.^21^ The sepsis and malnutrition in our patients were made worse by HIV infection.^4^, ^5^ Our patients had all risk factors associated with mortality in enterocutaneous fistulae namely sepsis, high output fistulae, fluid and electrolyte derangements, HIV infection mortality and hypoalbuminemia.^22^

**Figure 1:**
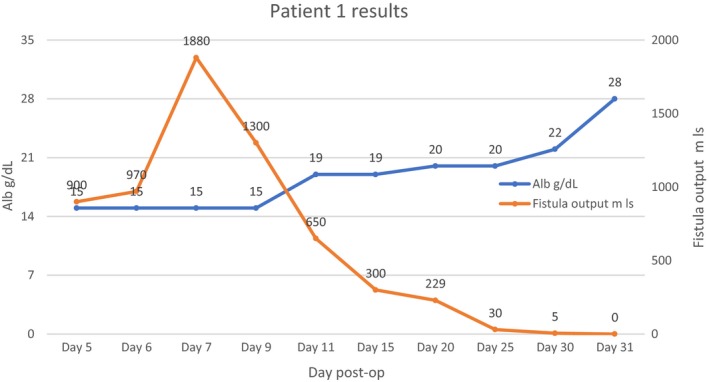
The relationship between albumin level and fistula output for patient 1

**Figure 2:**
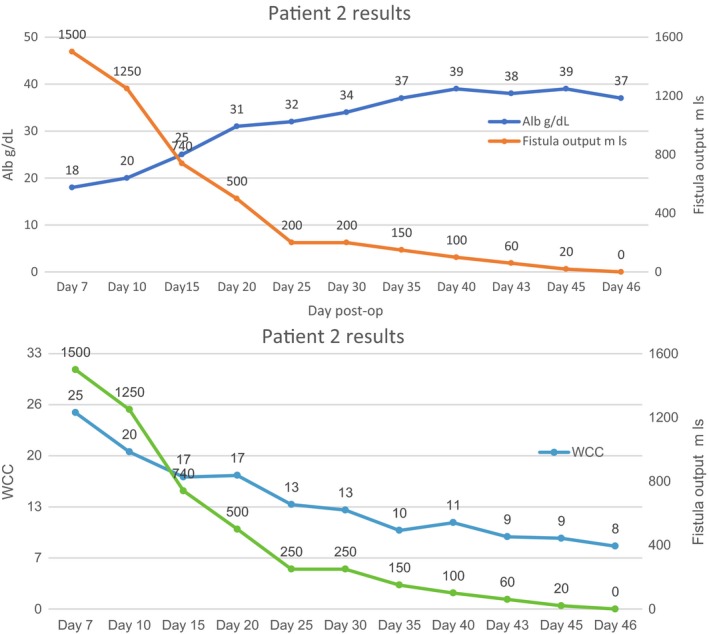
Shows the relationship between fistula output and; albumin (A), white cell count (WCC) (B) and for patient 2

**Figure 3:**
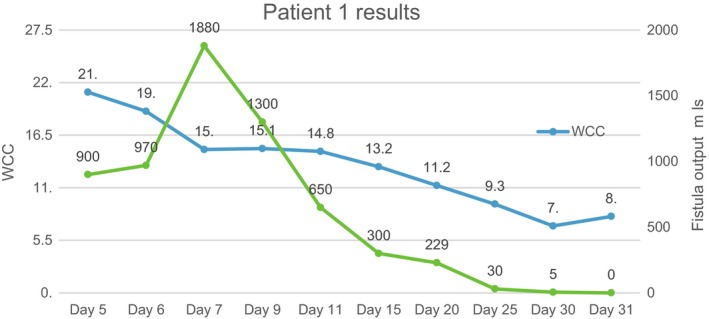
The relationship between white cell count and fistula output for patient 1

Early surgery which is advocated in patients with high output enterocutaneous fistula after stabilization could not be done in our patients in the presence of sepsis and malnutrition. We embarked on aggressive fluid resuscitation, correction of electrolyte abnormalities especially hypokalemia, control of sepsis, protection of skin and monitoring. The frequent evaluation of electrolytes allowed us to overcome the fluid and electrolyte challenge. Acid‐base balance evaluation and correction was another challenge we encountered. Our laboratory ran out of reagents to do blood gases during the duration of admission of the patients. This was only done once on the first case on admission. With the knowledge that high output ECF causes metabolic acidosis, we were able to go past this huddle by choosing the correct fluids for resuscitation and maintenance. Total parenteral nutrition (TPN) was not available at our institution and both patients could not afford the high price of this commodity from the private sector. This was another big challenge but with the knowledge that all patients with intestinal failure or ECF must be provided with some luminal source of nutrition to maintain enterocyte mass and prevent mucosal atrophy^23^ we continued to feed our patients enterally. Initially, there was a rebound in fistulae output to above 1000 mL/d for both patients. We then decided to give continuous enteral high protein high calorie/elementary nutrition via a nasogastric tube in small amounts. This was well tolerated than bolus feeds. There was a negative correlation between albumin and fistulae output in both patients as illustrated in Figures 1 and 2A. With a rise in albumin levels, there was a reduction in fistula output. In addition to the use of TPN, there is report of beneficial effects of percutaneous injection of cyanoacrylate glue in low output ECF without signs of complications or in patients unsuitable for surgery.^24^ This approach is easy and safe, viable, and useful for future trials on the efficacy in conservative treatment of EC fistula.^24^ Sepsis accounts for more than 70% of the mortality in patient with ECF.^10^ Sepsis in patients with enterocutaneous fistulae is mainly due to anaerobes, enterobacteria, and gram‐negative bacilli. All of our patients were put on Ceftriaxone 1 g twice daily and Metronidazole 500 mg 8 hourly intravenously for 3 weeks and thereafter Ciprofloxacin 500 mg twice a day orally when there was significant reduction in fistula output. This resulted in the control of sepsis and hastened fistula closure. As illustrated in Figures 2B and 3, there was a positive correlation between white cell count and fistula output. As the white cell count dropped to normal levels there was a decline in fistula output until nothing was being drained. Our two patients did not need surgery and were discharged on day 31 and 46.

## 5 CONCLUSION

Clinicians looking after HIV infected patients with high output ECF must give nonoperative management a chance especially in the presence of sepsis and malnutrition. It will eventually bring desired results. An anticipation must however be made that HIV infection would worsen the sepsis and malnutrition; hence, aggressive management and monitoring are advocated. In our two cases, correction of fluid and electrolyte abnormalities, control of sepsis, and malnutrition were positive predictors of fistulae closure.

## 6 CONSENT

Written informed consent was obtained from the patients for publication.

## CONFLICT OF INTEREST

None declared.

## AUTHOR CONTRIBUTION

DM: involved in the case report design, subject research, consent, editing and writing. EGM: involved in the case report design, subject research, editing and writing. SGM: involved in the case report design, subject research, editing and writing.

## ETHICAL APPROVAL

Ethical approval was exempted by our institution.
